# Effect of Breast Cancer Awareness Training on Screening Beliefs and Behaviour Among Women Living in Rural Areas of Türkiye: A Quasi-Experimental Study

**DOI:** 10.3390/healthcare14040531

**Published:** 2026-02-20

**Authors:** Gökhan İşçi, Zeliha Yelda Özer, Burak Mete, Çağla Okyar, Hakan Demirhindi

**Affiliations:** 1 Department of Family Medicine, Faculty of Medicine, Cukurova University, 01330 Adana, Türkiye; drgokhanisci@gmail.com (G.İ.); zyozer@cu.edu.tr (Z.Y.Ö.); 2 Department of Public Health, Faculty of Medicine, Cukurova University, 01330 Adana, Türkiye; demirhindi@cu.edu.tr; 3 Department of Medical Education and Informatics, Faculty of Medicine, Cukurova University, 01330 Adana, Türkiye; cokyar@cu.edu.tr

**Keywords:** breast cancer, screening, education, intervention study

## Abstract

Background/Objectives: This study aimed to evaluate the effect of breast cancer awareness training on screening beliefs and behaviour. Methods: This single-group pre-test/post-test quasi-experimental study included 286 women living in rural areas. The effectiveness of the educational intervention was evaluated via the Breast Cancer Screening Beliefs Scale (BCSBS). Screening behaviours were assessed using self-reported data collected via a structured questionnaire administered before the intervention. Results: The mean age of the participants was 58.3 ± 8.3 years (range 43–69). Post-test results showed a significant increase in the total BCSBS score and all sub-dimensions, including attitudes towards health screenings, breast cancer knowledge and perceptions, and barriers to mammography screening (*p* < 0.001). The educational intervention had a medium effect on screening beliefs with an effect size (ES) of 0.585, small effects on attitudes towards health screenings (ES = 0.124) and barriers to mammography screening (ES = 0.286), and a large effect on breast cancer knowledge and perceptions (ES = 0.710). Following the educational intervention, the proportion of women with positive screening beliefs increased from 13% to 36% (*p* < 0.001), positive breast cancer knowledge and perceptions increased from 49% to 69% (*p* < 0.001), and positive attitudes towards health screenings decreased from 14% to 8% (*p* = 0.002). Each one-unit increase in breast cancer knowledge and perceptions increased the likelihood of screening by an odds ratio (OR) of 1.02, and each one-unit increase in barriers to mammography screening increased it by an OR of 1.03. Moreover, barriers to mammography screening significantly moderated the relationship between age and breast cancer screening behaviour, strengthening this association. Conclusions: The Turkish Ministry of Health’s education model for breast cancer awareness appears to positively influence beliefs about breast cancer screening. Updating and diversifying educational content to target specific age groups and rural women may enhance its effectiveness.

## 1. Introduction

Breast cancer, the second most common cancer worldwide, is a significant public health problem and one of the leading causes of morbidity and mortality, and it is the most common cause of cancer deaths among women [1]. Breast cancer screening is an effective measure for detecting early-stage disease and improving survival rates among cancer patients [2,3]. Community-based breast cancer screening programmes implemented in many countries have contributed to reducing the mortality and incidence of advanced-stage cancer [4,5,6].

A combination of awareness, education, and screening activities is required to increase early diagnosis and improve outcomes. Awareness of both breast cancer itself and screening methods is essential to increase participation in screening programmes, which is impaired by a lack of knowledge both about breast cancer itself and its risks [7,8]. Furthermore, educational interventions targeting specific populations have been shown to improve knowledge and attitudes towards breast cancer screening, thereby increasing participation rates. This is particularly important for culturally and linguistically diverse communities, where tailored education programmes can effectively address barriers to screening [9].

The role of social determinants in breast cancer awareness and screening participation cannot be overlooked. Research shows that socioeconomically disadvantaged communities generally experience lower screening rates and higher rates of advanced-stage breast cancer at diagnosis [10]. Women with lower levels of education and income have been reported to be significantly less likely to participate in breast cancer screening programmes [11]. This highlights the need for targeted educational initiatives that take into account the challenges faced by these populations.

Given the tight-knit social structure of the rural community where this study was conducted, a single-group pre-test/post-test design was chosen to address both ethical and methodological concerns. From an ethical standpoint, withholding potentially life-saving health education from a control group within the same community was deemed inappropriate. Methodologically, the high probability of information contamination—the likelihood of participants sharing educational insights with their peers—posed a significant risk to the internal validity of a controlled design. Therefore, this study focused on evaluating the immediate impact of the intervention on a single cohort to ensure equitable access to health information while maintaining the study’s integrity. This study aimed to evaluate the effect of the Turkish Ministry of Health’s education model for breast cancer awareness training on beliefs about screening and screening participation behaviour among women living in a rural area in Adana, Türkiye, in the context of the following hypotheses:

**H_1_:**  *Participants will show a significant increase in breast cancer knowledge and perception scores following the intervention.*

**H_2_:**  *The intervention will lead to a significant decrease in perceived barriers to mammography screening.*

**H_3_:**  *There will be a significant increase in the participation rate in at least one screening behaviour.*

## 2. Materials and Methods

### 2.1. Research Type

This single-group pre-test/post-test quasi-experimental study was conducted in 2025 by researchers from the Department of Family Medicine at the Faculty of Medicine of Çukurova University, and included 441 women aged 40 to 69 years, registered at the Feke Central Family Health Centre—No 003 in the district of Feke in Adana province. The Academic Council of the Department of Family Medicine and the Ethics Committee for Non-interventional Research, both of the Faculty of Medicine at Çukurova University, granted the required permissions (dated 6 December 2024, with decision no. 150). Furthermore, the study was also evaluated and deemed appropriate by the Adana Provincial Health Directorate Scientific Research and Project Studies Evaluation Commission (dated 23 January 2025).

A single-group pre-test/post-test quasi-experimental design was employed for this study. This design was preferred over a randomised controlled trial primarily due to ethical and practical considerations. Within the small and tight-knit rural community where the study was conducted, withholding potentially life-saving breast cancer education from a control group was deemed ethically inappropriate. Additionally, the high risk of information contamination—where participants from different groups might share educational materials and insights—precluded the feasibility of a control group in this specific setting.

The temporal sequence of the intervention was strictly structured across three phases: (1) an initial baseline assessment (T1) to determine pre-existing knowledge and attitudes, (2) the immediate delivery of a standardised breast cancer awareness education programme, and (3) a follow-up assessment (T2) conducted exactly four weeks post intervention.

Regarding causal limitations, we acknowledge that the absence of a control group limits our ability to definitively attribute all observed changes solely to the intervention. Potential confounding factors, such as simultaneous public health campaigns or social interactions within the rural community, could influence the results. However, this design was selected due to the ethical and practical constraints of the study setting, as previously outlined.

Internal Validity: Several strategies were employed to mitigate potential threats to internal validity inherent in a single-group pre-test/post-test design. To minimise social desirability bias, participants were assured of the anonymity and confidentiality of their responses, and data collection was conducted by trained researchers who were not part of the community’s primary healthcare delivery team. To reduce testing effects (learning bias), the follow-up assessment was scheduled four weeks after the intervention—a sufficiently long period to prevent simple recall of baseline answers while remaining relevant to the intervention. Furthermore, the educational content was standardised and delivered using identical materials to all participants to ensure procedural consistency and minimise researcher-induced variability.

### 2.2. Sampling and Sample Size

There are 55 family health centres in the rural areas of Adana Province, and the Feke Family Health Centre region was determined using cluster sampling. There are 441 women aged 40–69 in the registered population. All women were contacted, and 310 women who agreed to participate in the study and met the inclusion criteria were included in the study.

Sample Size and Power: An a priori power analysis was not performed. Instead, a total population sampling strategy was adopted, aiming to include all eligible women residing in the targeted rural district during the study period. This approach was chosen to ensure the intervention reached as many community members as possible.

Written consent was obtained from all participants using an informed consent form.

Exclusion criteria:

- Having a current diagnosis of breast cancer or symptoms;
- Not speaking Turkish;
- Being diagnosed with dementia or severe psychiatric illness, or being bedridden.

**Figure 1:**
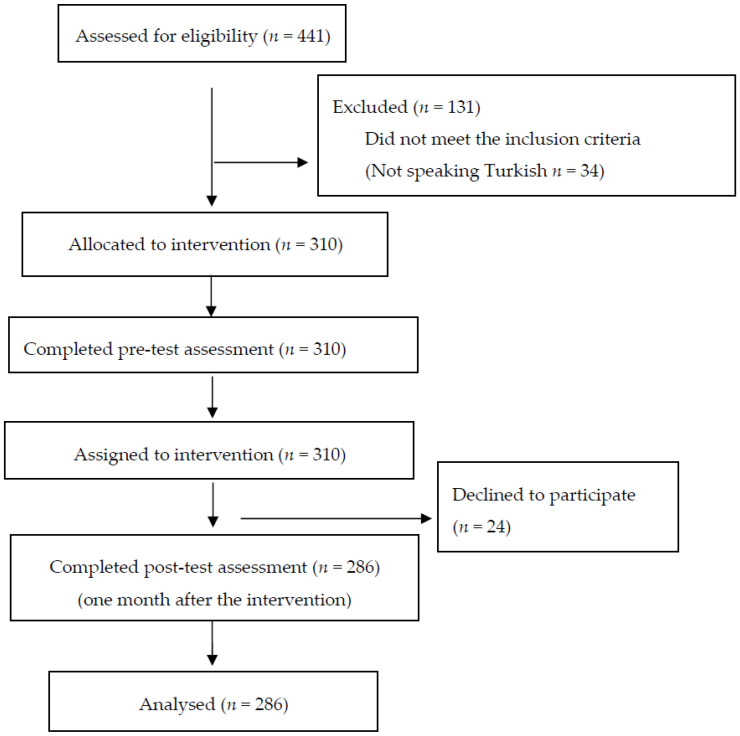
Flowchart.

Participants were not excluded based on their prior screening history. The study intentionally included both women who were non-adherent to screening guidelines and those who were already adherent. This inclusive approach was adopted to evaluate the impact of the educational intervention on the entire screening-eligible rural population, aiming to both initiate screening in new participants and reinforce correct practices among those already undergoing screening. While the study started with 310 women who participated in the first stage, it was completed with 286 women who participated in the second stage (Figure 1).

To minimise self-selection bias, the researchers collaborated with healthcare workers to reach a diverse range of women, including those who did not regularly attend local health centres.

### 2.3. Data Collection and Measurement Tools

- Sociodemographic Information Form (17 questions);
- Breast Cancer Screening Beliefs Scale;
- Educational videos on breast cancer screening provided by the Turkish Ministry of Health;
- Brochures on breast cancer as supporting educational material.

### 2.4. Educational Intervention

Data were collected using a face-to-face survey method after participants were informed about the study, and their written consent was obtained. Women were contacted at the family health centre unit if they were able to come to the centre. The women aged 40–69 who could not come were contacted during village visits and gathered in buildings where mobile health services were provided, after the event was announced by the local authority. Finally, those not contacted by either method were visited at their home.

The data collection process was carried out over the following two stages:

Stage 1: Participants were administered the “Sociodemographic Information Form” and the “Breast Cancer Screening Beliefs Scale” and were shown four short educational videos prepared by the Turkish Ministry of Health. The video content was as follows:
**Video Caption****Duration** **(Seconds)****Educational Content**What is mammography? [12]37One in every 8–10 women is at risk of developing breast cancer. Mammography is an imaging technique that uses very low levels of X-ray radiation to detect breast cancer in women at an early stage.Who should have a mammogram? [13]55Mammography can be performed for breast cancer screening in healthy women with no symptoms. It is recommended that women over the age of 40 attend screening once every two years. This service is available free of charge at the Heath Ministry’s “Early Diagnosis, Screening and Education Centres for Cancer (KETEM)”, “Community Health Centres” and “Healthy Life Centres”.Is having a mammogram risky? [14]76The pain and radiation exposure during a mammogram are minimal. Mammograms can save lives through early detection. Therefore, the potential risks are negligible.What should be considered before and during a mammogram? [15]69No special preparation is required for mammograms. However, if the scan is performed on pre-menopausal women, scheduling it according to the menstrual cycle may reduce pain or pressure sensation. Therefore, the ideal time for a mammogram is between the 7th and 14th day of the menstrual cycle. Before coming for the scan, it is important to avoid applying any external substances such as perfume or cosmetic products to the breast skin. This is because they can distort the image and cause blurring.

To ensure the standardisation and quality of the educational intervention, official multimedia training materials produced by the Turkish Ministry of Health were utilised. The choice of Ministry of Health (MoH) training videos over tailored materials was deliberate. These standardised videos provide an evidence-based, medically validated, and uniform educational framework that aligns with national cancer control policies. Furthermore, using MoH materials ensures the replicability of the intervention in other primary healthcare settings, as these resources are readily available to all healthcare providers in Türkiye, thus supporting the potential for wider public health impact.

Following the video, meetings were held with participants on breast cancer and the video content in a question-and-answer format.

Stage 2: Four weeks after the participants had watched the educational videos, the same “Breast Cancer Screening Beliefs Scale” was re-administered.

### 2.5. Breast Cancer Screening Beliefs (BCSB) Scale

The scale, which was developed by Kwok et al. in 2010 and adapted into Turkish in 2021 by Türkoğlu and Sis Çelik, consists of 13 items and three sub-dimensions [16,17]:

(1) Attitudes towards health screenings (items 1–4);
(2) Breast cancer knowledge and perceptions (items 5–8);
(3) Barriers to mammography screening (items 9–13).

The scale is scored using a five-point Likert scale, with each item of the original scale rated on a five-point Likert scale ranging from “strongly agree” (1-point score) to “strongly disagree” (5-point score). Item scores are converted to a range of 0 to 100 for analysis as follows: 1-point scores are converted to 0, 2-point ones to 25, 3-point ones to 50, 4-point ones to 75, and 5-point ones to 100. The sum of the converted scores was divided by the number of items in the sub-scales, yielding the mean score of the subscale. There are no reverse items on the scale. High scores indicate positive beliefs, and low scores indicate perceived barriers. The lowest possible score on the scale is 0, and the highest is 100. Subscale mean scores of 65 and above were found to be an indicator of an increase in screening beliefs, improved knowledge, and reduced barriers to mammography screening. The scale’s internal consistency coefficient (Cronbach’s Alpha) ranges from 0.76 to 0.87 [17].

### 2.6. Breast Cancer Screening Behaviour

Participation in screening was defined as engaging in at least one of the following breast cancer screening behaviours:

- For women over the age of 20, performing a self-breast examination once a month;
- For women aged 20–40, in addition to self-breast examinations, undergoing a routine clinical breast examination by a physician once a year for those who have a family history of breast cancer among first-degree relatives, or once every two years for those without such a history;
- For all women aged 41–69, undergoing a routine clinical breast examination by a physician once a year and a digital/conventional mammography once every two years.

Screening behaviours were assessed through self-reported data using a structured questionnaire administered before the intervention [18].

### 2.7. Statistical Analysis

The JAMOVI (2.7.15) software was used to analyse the data. The Shapiro–Wilk test was used as the normality test. Parametric tests were used to analyse data that followed a normal distribution, while non-parametric tests were used for non-normally distributed data. The Wilcoxon test, McNemar test, Chi-square test, logarithmic linear regression analysis, binary logistic regression analysis, and moderation analysis were used. Multiple logistic regression analysis was employed to identify the independent predictors of breast cancer screening behaviour, allowing for the simultaneous assessment of various psychological and demographic factors. Furthermore, moderation analysis was specifically selected to investigate whether the effectiveness of the educational intervention—expressed through changes in breast cancer beliefs—varied across different age groups. A *p* < 0.05 value was considered statistically significant.

## 3. Results

The mean age of the women included in the study was 58.3 ± 8.3 (min: 43–max: 69), 49% participants were in the 60–69 age group, the highest level of education was primary school (50%), and 92.3% were housewives. The rate of those who underwent breast cancer screening was 65.7%. The most important stated reason for not undergoing breast cancer screening was financial constraints. The vast majority (68.2%) had a monthly income below or at the national minimum wage in Türkiye. The prevalence of chronic disease was 34.3%, and the frequency of regular medication use was 36.0% (Table 1).

When comparing scores obtained from the BCSB scale before and after the intervention, statistically significant increases were observed in the median scores obtained from the total scale, and from the attitudes towards health screenings, breast cancer knowledge and perceptions, and barriers to mammography screening sub-scales. The effect size (ES) of the educational intervention on the BCSB scale’s total score was medium (ES = 0.585); meanwhile, it was small (ES = 0.124) on the sub-dimension of attitudes towards health screenings, large (ES = 0.710) on the sub-dimension of breast cancer knowledge and perceptions, and small (ES = 0.286) on the sub-dimension of barriers to mammography screening (Table 2).

When comparing the pre- and post-intervention BCSB scale scores of women by age group, no differences were found between the total scale score and the sub-dimensions of barriers to mammography screening before the training. However, after the training, it was found that the 40–49 age group had statistically significantly higher scores on the total scale (Table 3).

**Figure 2:**
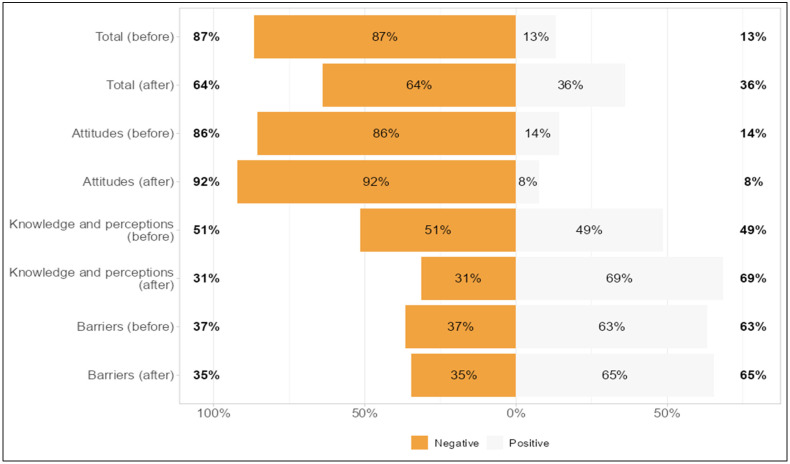
Score distributions before and after the intervention in the entire group.

When examining whether there was a positive increase in the BCSB scale’s sub-dimension scores before and after the intervention in all women (scale cut-off > 65), the positive rates in the overall scale assessment increased from 13% to 36% (*p* < 0.001), the positive rates in the sub-dimension of attitudes towards health screenings decreased from 14% to 8% (*p* = 0.002), the positive rates in the breast cancer knowledge and perceptions sub-dimension increased from 49% to 69% (*p* < 0.001), and the positive rates in the barriers to mammography screening sub-dimension increased from 63% to 65% (*p* = 0.522) (Figure 2).

**Figure 3:**
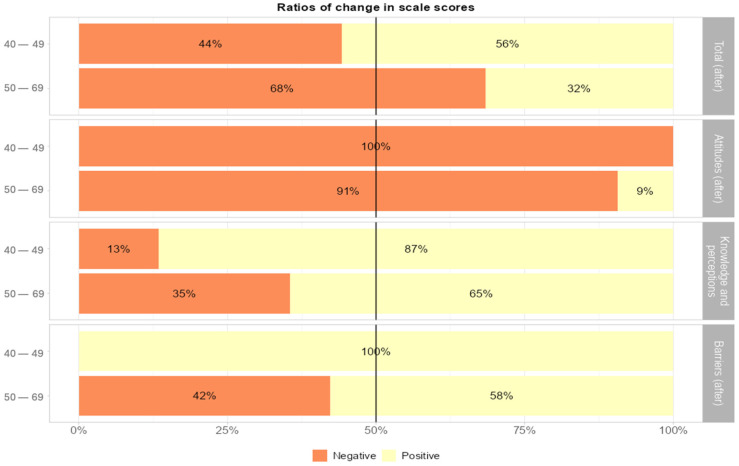
Change in scale sub-group scores by age group.

When comparing changes in scale sub-dimension scores following the intervention by age groups, the 40–49 age group showed positive changes in the scale’s total score (32% versus 56%; *p* < 0.001), in breast cancer knowledge and perceptions scores (87% versus 65%; *p* = 0.009), and in barriers to mammography screening scores (100% versus 58%; *p* < 0.001); with positive answers being statistically significantly higher in the 40–49 age group. In the sub-dimension of attitudes towards health screenings (9% vs. 0%), a greater increase in positive scores was found in the 50–59 age group (*p* = 0.002) (Figure 3).

Participants were grouped into two groups according to having or not having increased BCSB scale scores based on the difference between their pre- and post-intervention mean scores on the scale sub-dimensions, and interaction between age groups and score change was evaluated using a logarithmic linear regression model. The likelihood of a score increase for the 40–49 years age group was found to be 5.41 times higher for the total scale score, 2.68 times higher for the attitude towards health screenings sub-dimension, and 2.28 times higher for the barriers to mammography screenings sub-dimension (Table 4).

Values before and after the intervention were compared according to the history of participation in a breast cancer screening. Before the intervention, the median total score of individuals who had participated in a breast cancer screening was found to be significantly higher than that of those who had not participated. Similarly, after the intervention, the median total score of the group of women who had participated in screening was significantly higher than that of those who had not.

When attitudes towards general health check-ups were examined, the attitude scores of those who had participated in a screening before the intervention were significantly higher than the scores of those who had not. This difference was maintained after the intervention, and it was found that the median scores of individuals who had participated in a screening were significantly higher.

In terms of breast cancer knowledge and perception levels, no significant difference was observed between those who had participated in a screening before the intervention and those who had not, but after the intervention, it was determined that the knowledge and perception scores of individuals who had participated in a screening were significantly higher.

Similarly, when the sub-dimension scores of barriers to mammographic screening were evaluated, no significant difference was observed between the groups before the intervention. However, after the intervention, it was evident that the barrier perception scores of individuals who had participated in a screening differed significantly and positively, keeping in mind that a higher score in the ‘Barriers’ sub-dimension indicates a lower perception of obstacles.

Overall, the results showed that participation in breast cancer screening was closely related to general attitude, knowledge, and awareness levels, and that the intervention implemented led to significant improvements in these areas, particularly in the post-intervention period (Table 5).

The logistic regression analysis created to predict breast cancer screening behaviour, including pre-education BCSB scale sub-dimension scores, was found to be significant (*p* < 0.001) with good model fit (Nagelkerke R^2^ = 0.387). The variables breast cancer knowledge and perceptions, barriers to mammography screening, and age were found to contribute significantly to the model. Each unit increase in the breast cancer knowledge and perceptions sub-dimension increased the likelihood of undergoing screening by 1.02 times, while each unit increase in the barriers to mammography screening sub-dimension increased it by 1.03 times, and each unit increase in age increased the likelihood by 1.16 times (Table 6).

When changes occurring before and after the intervention were examined as having moderator roles in the relationship between age and breast cancer screening behaviour, it was found—specifically in the sub-dimensions of breast cancer beliefs, attitudes towards health screenings, breast cancer knowledge and perceptions, and barriers to mammography screening—that the sub-dimension of barriers to mammography screening was a significant moderator. A positive increase in the barriers to mammography screening sub-dimension—i.e., a lower perception of obstacles—was observed to strengthen the relationship between age and breast cancer screening (Table 7).

## 4. Discussion

Breast cancer is the most common cancer in women and one of the leading causes of death at a young age. The American College of Radiology recommends starting mammography screening at the age of 40 because it offers the possibility of earlier diagnosis, reduces mortality, and allows for better surgical options and more effective treatment. It has been proven that mammography screening in women aged 40 and over is effective at reducing breast cancer-related deaths and that regular screening can reduce mortality by up to 40% [19]. Despite the proven benefits of screening, participation levels have not reached the desired level. Our study evaluated the effect of the Turkish Ministry of Health’s breast cancer and screening awareness training on breast cancer screening beliefs and participation among women aged 40–69 living in rural areas. We found that the educational intervention was associated with positive effects in terms of attitudes towards health screenings, breast cancer knowledge and perceptions, and beliefs about barriers to mammography screening. The educational intervention was most effective in terms of breast cancer knowledge and perceptions, while smaller effects were observed in terms of attitudes towards health screening and barriers to mammography screening. After the intervention, positive belief rates increased in the breast cancer knowledge and perceptions sub-factor, but decreased in the attitude towards health screenings sub-factor. On the other hand, no significant change was observed in the barriers to mammography screening sub-factor. When effectiveness was assessed by age, it was found that the effectiveness of education was higher among women aged 40–49, and positive belief rates in this age group saw a greater increase.

Studies conducted in previous years reported numerous factors contributing to the low participation of women in breast cancer screening programmes in Türkiye, such as low educational level, insufficient knowledge about breast cancer and mammography, not being referred by a physician, lack of health insurance, family history, and health beliefs [20,21,22]. We found that, in the context of the BCSB scale, increased positive beliefs in the sub-scales of breast cancer knowledge and perception and barriers to mammography screening increased participation in screening. An increase in age also increased participation in screening, and the relationship between age and screening was strengthened by increased positive beliefs in the barriers to mammography screening subscale (which corresponds to a lower perception of obstacles). Despite the various strategies implemented, it is unclear which strategies are more effective in terms of improving breast cancer screening practices among women. However, the literature clearly indicates that more education is needed to increase the adoption of breast cancer screening practices [23,24].

Our study utilised educational videos prepared by the Turkish Ministry of Health, and it was observed that the training led to a positive increase in beliefs regarding breast cancer screening and that there was a positive correlation between positive beliefs and participation in screening. Overall, our results indicated that participation in breast cancer screening was closely related to general attitudes, knowledge, and perception levels, and that the intervention implemented generated significant improvements in these areas, particularly in the post-intervention period. A remarkable finding of this study was the decline in positive attitudes toward health screening following the intervention, particularly among participants in the 40–49 age group. This trend may be attributed to a ‘heightened anxiety effect’ stemming from increased awareness. Before the intervention, participants might have held a degree of ‘unrealistic optimism’ or a simplified perspective regarding screening procedures. However, the detailed educational content concerning the severity of breast cancer and the complexities of diagnostic procedures might have triggered temporary resistance or fear, leading to a decrease in positive predispositions. In contrast, the significant increase in positive scores within the 50–59 age group might be an indicator that the effectiveness of the intervention varied by age. This discrepancy points toward potential gaps in health literacy levels and suggests that the educational content resonated differently across various age cohorts. The decline observed in the 40–49 age group may suggest that the intervention may have inadvertently amplified risk-related anxiety rather than enhancing self-efficacy among younger middle-aged women. Future programmes should prioritise age-tailored messaging to strike a balance between risk awareness and psychological reassurance.

In a study by Canbulat et al. that evaluated the relationship between health beliefs and breast cancer screening behaviours in Türkiye, female healthcare workers who performed self-administered breast examinations were found to have significantly higher sensitivity perception (the level at which a person perceives him/herself to be at risk), health motivation, perceptions of the benefits of screening, and self-efficacy perceptions than those who did not. This finding shows that positive health beliefs are effective in encouraging breast screening among women [25]. In our study, it was also found that more positive beliefs regarding breast cancer screening knowledge and barriers to mammography were factors affecting screening behaviour. Furthermore, the higher rate of screening among older women (21.2% for ages 40–49 and 75.6% for ages 50–59) might be related to the lower perceived risk among younger women. Another factor explaining this situation was that the relationship between age and screening was strengthened by the increase in positive beliefs regarding barriers to mammography screening. The similarity in mammography barrier scores in the 40–49 and 50–59 age groups prior to the educational intervention, and the significantly higher scores in the 40–49 age group after the intervention, might be related to the lower perception of risk in the 40–49 age group. Based on our results, it can be said that the educational intervention contributed to a positive increase in beliefs about breast cancer screening among all women. However, when the sub-scales of the BCSBS were analysed by age group, the effect was greater in the relatively younger 40–49 age group, but participation in screening also remained lower. While participation in screening was higher in the 50–69 age group, the effect of the educational intervention was lower. These findings might be related to a lower perceived sensitivity among younger women and lower health literacy among older women. Considering that the participants in our study were women of low socioeconomic status living in rural areas, we can point to a need to diversify the educational content in a manner appropriate to the participants’ level of health literacy. Taking this context into account when preparing educational content and using more strategic educational models can increase the effectiveness of the training.

O’Mahony et al. compared two randomised controlled trials in their review. In the first study (867 women), participants were randomly assigned to receive a written brochure and routine care (intervention group 1), a written brochure and routine care plus verbal interaction with a radiologist or research psychologist (intervention group 2), or routine care alone (control group). In the second study (130 women), participants were randomly assigned to either an educational programme consisting of three sessions (each lasting 60–90 min) or a control group receiving no intervention. When the effectiveness of the studies was evaluated, knowledge about age-related breast cancer risk was reported to have increased in intervention group 1 compared to the control group in the first study, but this increase was not statistically significant. In intervention group 2, this increase was found to be significant. In the second study, perceived sensitivity increased significantly one month later in the group that had received training, while a decrease was observed in the control group. Breast cancer awareness in the first study did not change in intervention group 1 compared to the control group. In contrast, in intervention group 2, overall awareness increased significantly at the end of the two years. In the second study, a significant increase in awareness and perceived sensitivity scores was observed one month later in the group that had received the educational intervention, while this increase was lower in the control group. Based on the results of the study, it can be stated that short-term interventions have the potential to increase breast cancer awareness in women [26]. The results of our study also support the potential of increased breast cancer awareness and screening beliefs to lead to an increase in breast screening participation behaviour. In a review by Saei et al. involving 17,770 female participants, model-based educational interventions were shown to be more effective than non-model-based interventions in terms of self-administered breast examination, clinical breast examination, and mammography screening behaviours [27]. Structuring the Turkish Ministry of Health’s educational approach as a model-based intervention could increase the effectiveness of the intervention. Model-based educational interventions encourage self-care behaviours in women and form a basis for developing breast cancer screening behaviours. Furthermore, these interventions may increase policymakers’ awareness and efforts to improve breast cancer screening. A study by Günen et al. reported that women with high sensitivity perception according to the health belief model were more likely to use health screening methods [28]. Although the study was not primarily based on a specific behavioural theory, the results aligned with the constructs of the Health Belief Model (HBM), particularly regarding how the educational intervention modified ‘perceived barriers’ and ‘cues to action’ among women in rural areas.

In their study, Noman et al. found that the vast majority of studies examining the effectiveness of interventions reported positive results in terms of breast cancer screening participation, knowledge levels, and beliefs among women. Educational interventions appear to have the potential to increase breast cancer screening participation among women. However, it is important to emphasise that the findings should be interpreted with caution due to the marked heterogeneity among the studies in terms of participant characteristics, research designs, intervention strategies, and outcome measures [29]. Interpreting these results from another perspective, diversifying educational models according to social factors such as age and educational level may also increase the effectiveness of such interventions. Our results support this, indicating that educational programmes for less educated individuals should not only consist of knowledge transfer but should also encourage behavioural change.

## 5. Limitations and Strengths

There are some limitations to consider when interpreting the findings of our study. Firstly, the research was conducted among women registered at a single-family health centre in a rural area; this may limit the generalisability of the results to wider populations. Furthermore, selection bias might have occurred because individuals with higher health awareness were more likely to participate. The self-reported nature of the questionnaires might have led to information bias. A significant limitation of this study was the short follow-up period. The 4-week post-test timeframe might not be sufficient to observe long-term behavioural changes or a sustained commitment to breast cancer screening. Consequently, the results may primarily reflect immediate changes in awareness and intentions rather than permanent screening habits. The use of a single-arm pre-test/post-test design without a control group was a limitation of our study. This design might be susceptible to social desirability bias, where participants reported positive behaviours to meet perceived expectations, and regression to the mean, which could affect the statistical significance of the observed changes. The single-group pre-test/post-test design of this study was inherently subject to several internal validity threats. Beyond social desirability bias, the results might have been influenced by maturation (natural changes in participants’ health awareness over time) or history effects (external public health campaigns occurring simultaneously with our study). Additionally, the testing effect could not be ruled out, as completing the pre-test questionnaire might have sensitised participants to the topic, potentially inflating scores in the post-test assessment. These factors, inherent to quasi-experimental designs without a control group, necessitate a cautious interpretation of the intervention’s absolute effectiveness. Therefore, while our findings suggested an improvement in screening behaviours, they should be interpreted with caution and validated through future randomised controlled trials. On the other hand, a strength of our study was that it is one of the few studies in the literature using a quasi-experimental design and targeting a rural population.

## 6. Conclusions and Recommendations

Our study revealed that the breast cancer awareness education had a positive effect on the knowledge level, perceptions of breast cancer, and screening behaviours of women aged 40–69 in rural areas. Although the intervention was not primarily structured on a specific behavioural theory, the findings aligned with the Health Belief Model (HBM), demonstrating that education could effectively modify ‘perceived barriers’ and serve as a cue to action. However, the observed decline in positive attitudes among the 40–49 age group suggests that increased awareness might inadvertently trigger risk-related anxiety or resistance when not balanced with psychological reassurance. This highlights that a ‘one-size-fits-all’ educational model might be suboptimal due to the heterogeneous structure of society. To maximise effectiveness, future public health interventions should be age-tailored and region-specific, focusing on enhancing health literacy and self-efficacy while addressing the specific barriers of different life stages. Furthermore, incorporating longitudinal designs into future research will be essential to validating the permanence of the behavioural changes beyond the immediate post-intervention period.

## Figures and Tables

**Table 1:** Distribution of sociodemographic and breast cancer screening characteristics.

Variables	n (%)
Age	
40–49	52 (18.2)
50–69	234 (81.8)
Marital status	
Single	12 (4.2)
Married	273 (95.5)
Widow	1 (0.3)
Educational status	
Illiterate	90 (31.5)
Literate with no diploma	42 (14.7)
Primary school diploma	143 (50.0)
Secondary school diploma	11 (3.8)
Occupation	
Unemployed	11 (3.8)
Housewife	264 (92.3)
Civil servant	11 (3.8)
Household income	
Below or at the national minimum wage level	195 (68.2)
National minimum wage to the subsistence level	53 (18.5)
National subsistence level to poverty threshold	38 (13.3)
Family type	
Nuclear family	253 (88.5)
Extended family	9 (3.1)
Other	24 (8.4)
Chronic illness history	98 (34.3)
Psychological illness history	19 (6.6)
Family history of breast cancer	11 (3.8)
Breast cancer cases in the area where they reside	9 (3.1)
Participated in breast cancer education	11 (3.8)
Participated in breast cancer screening	188 (65.7)
Reason for not undergoing breast cancer screening Socioeconomic constrains	90 (91.9)
Transportation issues	8 (8.1)
Would undergo screening if a mobile screening vehicle were available	273 (95.5)
Total	286 (100)

**Table 2:** Comparison of Breast Cancer Screening Beliefs Scale scores before and after the educational intervention.

			Percentiles			
Sub-Dimensions of the Scale	Median	IQR	25th	75th	* p *	Effect Size **
Attitudes towards general health check-ups (before)	18.8	37.5	0.00	37.5	0.139	0.124
Attitudes towards general health check-ups (after)	25.0	54.7	1.56	56.3		
Knowledge and perceptions about breast cancer (before)	62.5	43.8	37.5	81.3	<0.001 *	0.710
Knowledge and perceptions about breast cancer (after)	81.3	37.5	62.5	100.0		
Barriers to mammographic screening (before)	70.0	20.0	60.0	80.0	<0.001 *	0.286
Barriers to mammographic screening (after)	80.0	15.0	65.0	80.0		
Total scale score (before)	55.8	25.0	38.46	63.5	<0.001 *	0.585
Total scale score (after)	63.5	30.8	46.15	76.9		

* Statistically significant values; ** rank-biserial correlation (Wilcoxon); IQR, inter-quartile range.

**Table 3:** Comparison of the Breast Cancer Screening Beliefs Scale scores before and after the educational intervention by age groups.

Sub-Dimensions of the Scale	Age	Median	IQR	* p *
Attitudes towards general health check-ups (before)	40–49	25.0	25.0	0.099
	50–69	18.8	37.5	
Attitudes towards general health check-ups (after)	40–49	50.0	56.3	0.846
	50–69	18.8	50.0	
Knowledge and perceptions about breast cancer (before)	40–49	81.3	12.5	<0.001 *
	50–69	56.3	43.8	
Knowledge and perceptions about breast cancer (after)	40–49	100.0	18.8	<0.001 *
	50–69	75.0	37.5	
Barriers to mammographic screening (before)	40–49	70.0	25.0	0.536
	50–69	70.0	20.0	
Barriers to mammographic screening (after)	40–49	80.0	0.0	<0.001 *
	50–69	80.0	20	
Total scale score (before)	40–49	51.9	13.5	0.893
	50–69	57.7	25.0	
Total scale score (after)	40–49	76.9	25.0	0.004 *
	50–69	62.5	32.7	

* Statistically significant values; IQR, inter-quartile range.

**Table 4:** Logarithmic linear regression analysis of score increases and age interaction.

						95% Confidence Interval	
Predictor	Estimate	SE	Z	* p *	Rate Ratio	Lower	Upper
Model 1							
Age ∗ Total mean difference group							
(40–49—50–69) ∗ (Increase—No change)	1.689	0.4270	3.96	<0.001	5.41	2.34	12.50
Model 2							
Age ∗ Attitude difference group							
(40–49—50–69) ∗ (Increase—No change)	0.988	0.3139	3.15	0.002	2.68	1.45	4.96
Model 3							
Age ∗ Knowledge difference group							
(40–49—50–69) ∗ (Increase—No change)	−0.441	0.314	−1.41	0.160	0.64	0.34	1.19
Model 4							
Age ∗ Barriers difference group							
(40–49—50–69) ∗ (Increase—No change)	0.825	0.3197	2.58	0.010	2.28	1.21	4.26

SE, standard error. ∗: the interaction term in the statistical model.

**Table 5:** Comparison of pre- and post-intervention Breast Cancer Screening Belief scale scores according to having a history of participation in breast screening.

	Breast Cancer Screening History	Median	IQR	* p *
Attitudes towards general health check-ups (before)	No	0.0	25.0	<0.001 *
	Yes	25.0	31.3	
Attitudes towards general health check-ups (after)	No	18.8	50.0	0.005 *
	Yes	25.0	50.0	
Knowledge and perceptions about breast cancer (before)	No	68.8	37.5	0.231
	Yes	62.5	43.8	
Knowledge and perceptions about breast cancer (after)	No	68.8	43.8	<0.001 *
	Yes	87.5	37.5	
Barriers to mammographic screening (before)	No	70.0	25.0	0.148
	Yes	70.0	15.0	
Barriers to mammographic screening (after)	No	70.0	15.0	0.001 *
	Yes	80.0	15.0	
Total scale score (before)	No	46.2	25.5	<0.001 *
	Yes	61.5	21.2	
Total scale score (after)	No	53.8	36.5	0.001 *
	Yes	63.5	32.7	

* Statistically significant values; IQR, inter-quartile range.

**Table 6:** Logistic regression analysis predicting breast cancer screening behaviour.

						95% Confidence Interval			
Predictor	Estimate	SE	Z	*p*	Odds Ratio	Lower	Upper	VIF	Tolerance
Intercept	−11.66	1.643	−7.10	<0.001	8.59 × 10^−6^	3.42 × 10^−7^	2.15 × 10^−4^		
Attitudes towards general health check-ups (before)	−0.007	0.006	−1.23	0.217	0.993	0.981	1.00	1.47	0.679
Knowledge and perceptions about breast cancer (before)	0.019	0.006	2.94	0.003 *	1.020	1.007	1.03	1.54	0.651
Low barriers to mammographic screening (before)	0.03	0.011	2.84	0.004 *	1.033	1.010	1.06	1.15	0.868
Age	0.15	0.021	7.28	<0.001 *	1.168	1.120	1.22	1.18	0.844

*, statistically significant, SE, standard error, VIF, variance inflation factor.

**Table 7:** Moderator effect of breast cancer beliefs.

Predictor	Moderator	Dependent Variable	Interaction *p*	Beta		
				Average	Low (−1SD)	High (+1SD)
Age	Attitudes towards general health check-ups	Screening	0.502	0.027	0.029	0.024
Age	Knowledge and perceptions about breast cancer	Screening	0.551	0.026	0.024	0.028
Age	Barriers to mammography screening	Screening	<0.001	0.025 *	0.007	0.042 *

Interaction = Predictor × Dependent variable; *, statistically significant.

## Data Availability

The data presented in this study are available upon request from the corresponding author but may be withheld due to ethical restrictions.

## Abbreviations

The following abbreviations are used in this manuscript:

BCSB	Breast Cancer Screening Beliefs
BCSBS	Breast Cancer Screening Beliefs Scale
KETEM	Early Diagnosis, Screening and Education Centres for Cancer
